# Case presentation-3

**DOI:** 10.1192/j.eurpsy.2026.10270

**Published:** 2026-07-31

**Authors:** S. Medved

**Affiliations:** University Clinical Hospital Zagreb, Zagreb, Croatia

## Abstract

**Abstract:**

Trauma-related disorders affect a significant portion of the population. Among others, they include post-traumatic stress disorder (PTSD), acute stress disorder and adjustment disorders. Some exposed to trauma will develop PTSD and other conditions such as anxiety, depression and substance use disorder. Those conditions are seen in everyday clinical practice and are expected to increase due to ongoing social issues and conflicts. We will present a case study of a patient that reflects current challenges and possibilities in trauma-related disorders.

**Disclosure of Interest:**

None Declared

